# Gram-Negative Bacterial Lipopolysaccharide Promotes Tumor Cell Proliferation in Breast Implant-Associated Anaplastic Large-Cell Lymphoma

**DOI:** 10.3390/cancers13215298

**Published:** 2021-10-22

**Authors:** Maria Mempin, Honghua Hu, Karen Vickery, Marshall E. Kadin, H. Miles Prince, Nicola Kouttab, John W. Morgan, William P. Adams, Anand K. Deva

**Affiliations:** 1 Surgical Infection Research Group, Faculty of Medicine, Health and Human Sciences, Macquarie University, Sydney, NSW 2109, Australia; maria.mempin@mq.edu.au (M.M.); karen.vickery@mq.edu.au (K.V.); 2 Warren Alpert School of Medicine, Brown University, Providence, RI 02903, USA; marshall_kadin@brown.edu; 3 Epworth Healthcare, Peter MacCallum Cancer Center, Melbourne, VIC 3000, Australia; miles.prince@petermac.org; 4 Department of Oncology, University of Melbourne, Melbourne, VIC 3001, Australia; 5 Roger Williams Medical Center, Providence, RI 02908, USA; nkouttab@gmail.com (N.K.); jmorgan@chartercare.org (J.W.M.); 6 Department of Plastic Surgery, University of Texas Southwestern, Dallas, TX 75205, USA; wpajrmd@dr-adams.com; 7 Integrated Specialist Healthcare Education and Research Foundation, Sydney, NSW 2228, Australia

**Keywords:** breast implant-associated anaplastic large-cell lymphoma, lipopolysaccharide, tumor cell, proliferation, T-cells’ malignancy

## Abstract

**Simple Summary:**

The development of a rare cancer of the immune system (lymphoma) associated with breast implants has been increasingly reported around the world. It has been hypothesized that the cancer is triggered by inflammation from bacteria residing within the textured surface of these implants, transforming the lymphocytes of some genetically prone patients over many years. This study shows that bacteria rather than the implant itself can trigger activation and multiplication of these cancer cells in the laboratory, lending support that bacteria and their products play an important role in causation. The unique response of these cancer cells to bacterial antigen was dampened significantly in the presence of a Toll-like receptor 4 inhibitor peptide. This finding has significance for both cancer prevention and treatment.

**Abstract:**

Breast implant-associated anaplastic large-cell lymphoma (BIA-ALCL) is a distinct malignancy associated with textured breast implants. We investigated whether bacteria could trigger the activation and multiplication of BIA-ALCL cells in vitro. BIA-ALCL patient-derived BIA-ALCL tumor cells, BIA-ALCL cell lines, cutaneous ALCL cell lines, an immortal T-cell line (MT-4), and peripheral blood mononuclear cells (PBMC) from BIA-ALCL, capsular contracture, and primary augmentation patients were studied. Cells were subjected to various mitogenic stimulation assays including plant phytohemagglutinin (PHA), Gram-negative bacterial lipopolysaccharide (LPS), Staphylococcal superantigens enterotoxin A (SEA), toxic shock syndrome toxin-1 (TSST-1), or sterilized implant shells. Patient-derived BIA-ALCL tumor cells and BIA-ALCL cell lines showed a unique response to LPS stimulation. This response was dampened significantly in the presence of a Toll-like receptor 4 (TLR4) inhibitor peptide. In contrast, cutaneous ALCL cells, MT-4, and PBMC cells from all patients responded significantly more to PHA, SEA, and TSST-1 than to LPS. Breast implant shells of all surface grades alone did not produce a proliferative response of BIA-ALCL cells, indicating the breast implant does not act as a pro-inflammatory stimulant. These findings indicate a possible novel pathway for LPS to promote BIA-ALCL cell proliferation via a TLR4 receptor-mediated bacterial transformation of T-cells into malignancy.

## 1. Introduction

Breast implant-associated anaplastic large-cell lymphoma (BIA-ALCL) is a recently recognized distinct malignancy of T lymphocytes associated with textured breast implants used for both aesthetic and reconstructive surgery [1,2,3,4]. Its incidence is increasing worldwide [2,3]. We previously put forward a unifying hypothesis implicating a combination of high surface area textured implants, bacterial contamination, genetic susceptibility, and time of exposure to explain its pathogenesis [3]. This hypothesis is supported by laboratory evidence (higher growth of bacteria on textured implants both in vitro and in vivo [5], linear increase in lymphocyte activation proportional to bacterial load [6], detection of bacterial species with shift in the microbiome towards the Gram-negative spectrum in BIA-ALCL specimens [7], accumulation of JAK1 and STAT3 mutations in patients with BIA-ALCL [8]) and epidemiological evidence (up to 23 times higher risk of BIA-ALCL for implants with high surface area that supports higher rates of bacterial growth in vivo [3,9]).

The development of BIA-ALCL is likely to be a complex process resulting from an interplay of host, implant, and microbial factors, including the patient’s genetic background, immune response, the textured implant surface, and bacterial phenotype that leads to neoplastic lymphoid tissue progression. This could account for why some patients with biofilm infection around breast implants proceed to contracture and why others, although less common, proceed to lymphocytic hyperplasia and BIA-ALCL.

Given increasing evidence around the epidemiology of BIA-ALCL that bacterial presence acts as a significant pro-inflammatory transformative driver, we aimed to investigate whether Gram-negative and Gram-positive bacterially derived antigenic drivers would interact differentially with BIA-ALCL tumor cells. We stimulated control and tumor cells with a plant-derived non-specific mitogen (Phytohemagglutinin (PHA)) and various bacterial antigens, including Gram-negative bacterial lipopolysaccharide (LPS), Gram-positive Staphylococcal superantigens Enterotoxin A (SEA), and Toxic Shock Syndrome Toxin-1 (TSST-1), since their role and potential to restrict T-cell receptor expression has been reported [10] and both *Staphylococcus aureus* and coagulase negative Staphylococci are frequently isolated from biofilms surrounding medical implants [11]. PHA was used as a control for a non-bacterial mitogen stimulation of tumor cells. PHA is a lectin extract from the red kidney bean (*Phaseolus vulgaris*) and contains potent, cell agglutinating, and mitogenic activities and activates normal T-cells by binding to cell membrane glycoproteins, including the T-cell receptor (TCR)-CD3 complex [12].

LPS is an endotoxin, forming about 75% of the outer membrane of Gram-negative bacteria [13]. The structure of LPS consists of a hydrophobic lipid A domain, an oligosaccharide core, and the outermost O-antigen [13]. Lipid A can be recognized by the innate immune system and causes macrophage activation and release of pro-inflammatory cytokines, with small doses capable of producing lethal shock [14]. The O-antigen, on the other hand, interacts with the adaptive immune system [14]. LPS and its lipid A moiety stimulate host cells via the Toll-like receptor (TLR) 4. TLR4, also known as CD284, is a member of the TLR protein family, part of the innate immune system, which recognizes common pathogen-associated molecular patterns [15]. Stimulation by LPS results in the generation of various proinflammatory cytokines, such as tumor necrosis factor-alpha (TNF-α), interleukin (IL)-1, and IL-6 [15].

SEA from *S. aureus* stimulates proliferation of peripheral lymphocytes, induces the production of interferons, and is important for gut immunity against *S. aureus* infections [16]. TSST-1, responsible for toxic shock syndrome, is secreted by *S. aureus* in response to environmental stress, such as low oxygen tension or low nutrient content in its surroundings [17]. It activates production of immune signaling molecules such as TNF-α, IL-1, M protein, and IFN-γ [17].

In this study, we investigated whether bacterially derived antigenic drivers would interact differentially with BIA-ALCL tumor cells as compared with tumor cells derived from other lymphomas and with peripheral blood mononuclear cells (PBMC) harvested from patients with BIA-ALCL, from patients having breast implants removed due to capsular contracture, and from healthy control subjects without exposure to breast implants. Texturing of the breast implant surface shell is one of the recurring features in BIA-ALCL cases and has been proposed as a cause of BIA-ALCL. Therefore, we also investigated if various implant shells could promote BIA-ALCL proliferation.

TLR play an important role in immunosurveillance and responses towards commensal and pathogenic microorganisms [18]. Although the link between TLR, Gram-negative bacteria, and inflammation is well known [19], the association between TLR and BIA-ALCL is still unknown. We also sought to compare TLR expression of tumor cells and to investigate the dampening of any observed proliferative response of tumor cells using chemical inhibitors of the TLR pathway to explore the possible mechanisms/pathway of LPS-mediated T-cells’ transformation into malignancy.

## 2. Materials and Methods

### 2.1. Tumor Cells, Peripheral Blood Mononuclear Cells, Cell Lines

Patient-derived BIA-ALCL tumor cells, BIA-ALCL cell lines, cutaneous ALCL cell lines, an immortal T-cell line (MT-4), and peripheral blood mononuclear cells (PBMC) from BIA-ALCL, contracture, and primary augmentation patients were studied.

#### 2.1.1. BIA-ALCL Tumor Cells, Cell Lines

Sixteen clinically diagnosed BIA-ALCL Australian patients presenting with a unilateral malignant effusion (seroma) or tumor mass were included in this study (Table A1). The seroma fluid was collected by puncture and aspiration under sterile conditions. The seroma fluid or tumor mass was kept on ice during transport to the laboratory for immediate analysis.

BIA-ALCL tumor cells were recovered fresh from malignant effusion and/or mass by centrifugation at 400 g for 5 min at 22 °C. The recovered tumor cells were histologically evaluated and confirmed CD30-positive by immunohistochemistry.

Three established BIA-ALCL cell lines, T-cell breast lymphoma (TLBR)-1, -2, and -3 [20], were used. These were derived from women aged between 42 and 45 who presented with seromas after exposure to either Allergan Biocell or Nagor breast implants (Table A2).

#### 2.1.2. Cutaneous ALCL Cell Lines

Two clonally related CD30+ ALK- cutaneous ALCL cell lines, MAC-1 and MAC-2A, derived from successive steps during progression of lymphomatoid papulosis to primary cutaneous ALCL, were utilized [21,22].

#### 2.1.3. MT-4 Cell Line

MT-4 cell line (ECACC Ref No.: 08081402, Salisbury, United Kingdom) was derived from a 50-year-old Japanese male with adult T-cell leukemia. The cells carry human T-lymphotropic virus-1 and support the growth of human immunodeficiency virus [23,24].

#### 2.1.4. Peripheral Blood Mononuclear Cells (PBMC)

Peripheral blood was obtained from patients with BIA-ALCL (*n* = 5), established grade IV capsular contracture (*n* = 3), patients undergoing primary cosmetic augmentation prior to exposure to breast implants (*n* = 3), and from one patient with early breast seroma. Peripheral blood was collected fresh in ethylenediaminetetraacetic acid tubes (BD Biosciences, Franklin Lakes, NJ, USA) and sent directly to the laboratory for immediate processing. PBMC were isolated from whole blood using Ficoll Hypaque density gradient centrifugation (GE Healthcare Life Sciences, Piscataway, NJ, USA).

#### 2.1.5. Cell Culture Conditions

BIA-ALCL and cutaneous ALCL cell lines were grown in Dulbecco’s Modified Eagle Medium (DMEM, Sigma-Aldrich, St. Louis, MO, USA) supplemented with 10% fetal bovine serum (FBS, Sigma-Aldrich) and 100 U/mL penicillin and 100 μg/mL streptomycin (Sigma-Aldrich). MT-4 cells and PBMC were grown in RPMI 1640 medium (Sigma-Aldrich) with the same supplements. All cell incubations were at 37 °C, 5% CO_2_.

#### 2.1.6. Ethics Statement

This study was approved by Macquarie University human ethics committee (Reference No. 5201600427). Informed consent was obtained from all participating patients.

### 2.2. Cell Proliferation Assays

Phytohemagglutinin (PHA), lipopolysaccharide (LPS), Staphylococcal enterotoxin A (SEA), and toxic shock syndrome toxin-1 (TSST-1) (Sigma-Aldrich) were reconstituted in RPMI 1640 medium at 11 times the desired final concentration, filter sterilized, and stored in aliquots at −30 °C, as described previously [25]. Each mitogen/antigen was used at final in-use concentrations of 5 and 10 µg/mL.

Cells (200 µL) were seeded at 1 × 10^5^ cells/mL, 5 × 10^5^ cells/mL, and 1 × 10^6^ cells/mL into six replicate wells of a 96-well cell culture plate. Cells were stimulated to proliferate non-specifically for 72 h, with 20 µL of mitogen/antigen, while control unstimulated cells received 20 µL of complete medium. Cell proliferation was measured using an MTT (3-[4,5-dimethylthiazol-2-yl]-2,5-diphenyl tetrazolium bromide) colorimetric assay (Roche, Basel, Switzerland). Briefly, 20 µL of MTT labeling reagent (5 mg/mL in PBS) was added to each well and incubated for 6 h. The culture supernatant was then discarded, 10% SDS in 0.01 M HCl was added, and the amount of MTT formazan produced was measured at a wavelength of 570 nm. Cell proliferation was expressed as a stimulation index (SI), calculated as follows:

$$Stimulation\;Index\;\left(SI\right)=\frac{OD_{570}\;of\;stimulated\;cells}{OD_{570}\;of\;unstimulated\;cells}$$

### 2.3. Cell Viability and Apoptosis Assays

BIA-ALCL (*n* = 11) and TLBR (*n* = 3) cell viability and presence of apoptosis was determined using Zombie UV fixable viability dye (Biolegend Cat No.: 423107, San Diego, CA, USA) and FITC Annexin V Apoptosis Detection Kit (BD Biosciences Cat No.: 556547), respectively. Annexin V positivity precedes the loss of the plasma membrane, which indicates early-stage apoptosis that will lead to cell death as detected by positive Zombie UV staining.

Cells (1 × 10^6^) were stimulated with 10 μg/mL of LPS or PHA for 72 h, washed three times, and incubated with 1 μL of Zombie UV dye and 5 μL of FITC Annexin V in 500 μL binding buffer for 15 min at room temperature (RT) in the dark. Cells were then washed with binding buffer, pelleted, fixed in 150 μL of 4% paraformaldehyde (ProSciTech, Thuringowa, QLD, Australia) for 15 min at RT, washed in PBS, and pelleted. The cell pellet was resuspended in 500 μL of PBS and filtered through a 35-μm filter tube to remove debris. Then, 50 μL of a known concentration of counting beads (BD Biosciences Cat No.: 349480) were added prior to flow cytometry (LSR Fortessa X-20 flow cytometer, BD Biosciences). FlowJo software (Tree Star Inc., Ashland, OR, USA) was used for data analysis. At least 10,000 live events were acquired. General gating strategy included forward scatter (FSC) and side scatter (SSC) to exclude cell debris, and forward scatter area (FSC-A) and height (FSC-H) to exclude doublets. Counting beads were gated on their intense fluorescence signal in the FSC-A vs. SSC-A scatter plot. Unstained and single-stained control samples were included with each experiment. To calculate the number of events corresponding to each apoptotic stage, specific cell populations were gated on a bivariate dot plot corresponding to the expression of Zombie UV versus Annexin V-FITC. Analysis of viable and apoptotic cells included live cells (Annexin V-negative, Zombie UV-negative) and both early apoptotic (Annexin V-positive, Zombie UV-negative) and dead cells (Annexin V-negative, Zombie UV-positive) plus late apoptotic/necrotic cells (Annexin V-positive, Zombie UV-positive). Calculation of absolute cell numbers for each sample was performed using the following formula:

$$\frac{number\;of\;events\;in\;cell\;region}{number\;of\;events\;in\;bead\;region}\times \frac{number\;of\;beads/test}{test\;volume}\times dilution\;factor\;$$

### 2.4. Effect of Implant Surface Texture on BIA-ALCL Cell Proliferation

The effect of implant textures of varying grades (Table A3) on BIA-ALCL tumor cell proliferation was determined in the presence or absence of LPS stimulation. Contaminating silicone was removed from implants, prior to obtaining 5-mm diameter sections using a punch biopsy tool (Kai Industries Co., Ltd., Seki, Gifu, Japan). Sections were then dry heat sterilized at 115 °C for 39 h.

BIA-ALCL cells (1 × 10^6^ cells/mL) from a single patient were seeded at a volume of 500 μL in six replicate wells of a 24-well cell culture plate and the following conditions were tested: (1) BIA-ALCL cells only, (2) BIA-ALCL cells + implant shell of varying grades (with the outer surface in contact with the cells), (3) BIA-ALCL cells + 10 μg/mL of LPS, and (4) BIA-ALCL cells + implant shell + 10 μg/mL of LPS. Plates were incubated at 37 °C for 72 h and SI was determined as above.

### 2.5. TLR4 Detection Assay

Expression of TLR4 in the three BIA-ALCL cell lines (TLBR-1, -2, -3) was examined by flow cytometry using anti-human CD284 (TLR4; BioLegend, San Diego, CA, USA). The human TLR4 stable cell line (Novus Biologicals; NBP2-26268, Littleton, CO, USA), expressing full-length human TLR4 with an N-terminal hemagglutinin (HA) tag, was used as a positive control. The TLR4 cell line was grown in DMEM with 10% FBS ± Blasticidin and all cell lines were grown with or without LPS. TLBR and control cell lines were stained with a Zombie NIR fixable viability dye and PE-conjugated anti-TLR4 and then fixed. Fixed cells were permeabilized using the BD Cytofix/Cytoperm kit (BD Biosciences) and stained intracellularly with anti-TLR4. Flow cytometry was performed on a Becton Dickinson CyAn ADP flow cytometer (BD Biosciences).

### 2.6. TLR4 Inhibition on Cell Proliferation and TNF-α Production Assays

Seven patient-derived BIA-ALCL tumor cells and two BIA-ALCL cell lines (TLBR-2 and -3) were seeded at 1 × 10^6^ cells/mL into triplicate wells (200 µL) of a 96-well plate pre-filled with 20 µL of 30 μM TLR4 inhibitor peptide (VIPER; Novus Biologicals, NBP2-26244) or control peptide (CP7; Novus Biologicals, NBP2-31231). The plate was incubated for 2 hours prior to addition of 10 μg/mL of LPS for test cells or 20 μL of complete DMEM for control unstimulated cells and cells were incubated for a further 72 h. After that, the inhibition of LPS-induced TLR4 activation by VIPER on cell proliferation was measured using the MTT assay, as described in Section 2.2. The cell culture supernatants were also collected to quantitate LPS-induced TNF-α secretion using an enzyme-linked immunosorbent assay (ELISA; Novex^®^, ThermoFisher Scientific, Waltham, MA, USA) following the manufacturer’s protocol. The analytical sensitivity of the assay is 1.7 pg/mL human TNF-α and is specific enough to avoid cross-reactivity of other recombinant cytokines.

### 2.7. Statistical Analysis

All statistical analyses were performed using GraphPad Prism version 8 (GraphPad Software Inc., San Diego, CA, USA). The Shapiro–Wilk test was used to check that data were normally distributed. The one-way or two-way analysis of variance (ANOVA) Kruskal–Wallis test by ranks, the Mann–Whitney rank sum test and Tukey’s or Sidak’s multiple comparisons post hoc tests were used to evaluate cell proliferation responses after mitogenic stimulation in the presence or absence of various implant shell textures, the differences in cell viability and apoptosis among BIA-ALCL tumor cells, and the differences in LPS-induced TNF-α production and proliferation responses of BIA-ALCL tumor cells. *p* values less than 0.05 were considered statistically significant.

## 3. Results

### 3.1. Patients’ Clinical Features

Confirmation of BIA-ALCL was based on immunohistochemical/flow cytometry findings by a clinical pathologist and included pleiomorphic cells being CD3+, CD4+, CD30+, and anaplastic lymphoma kinase protein negative (ALK−). Clinical data and breast implant type, from each of the 16 BIA-ALCL patients, are listed in Table A1. PBMC (*n* = 5) were also purified from these patients. The mean patient age was 43.8 years (range, 29 to 58 years) and the mean duration of time between insertion of implants and diagnosis of BIA-ALCL was 7 years (range, 0.1 to 20 years). In three patients, the indication for breast implants was post-mastectomy reconstruction (19%) and in the remaining patients, cosmetic augmentation (81%). Fifteen patients (94%) presented with a unilateral malignant effusion, whereas patient number 1627 presented with a tumor mass following infection. In two patients (1626 and 1714) the diagnosis of BIA-ALCL was preceded by capsular contracture. All patients were exposed to textured implants and upon diagnosis were treated with capsulectomy and removal of implants.

PBMC were collected from three capsular contracture patients, aged 42, 58, and 62 years, who had Silimed polyurethane, Allergan Biocell, and Mentor Siltex textured implants, respectively. PBMC were also collected from three healthy controls, with no implant exposure, aged 35, 37, and 39 years.

### 3.2. Cell Proliferation Response to Mitogenic/Antigenic Stimulation

**Figure 1:**
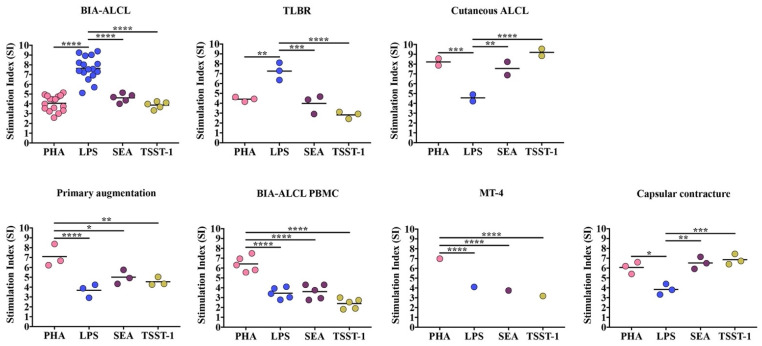
Maximum proliferative response as stimulation index (SI) of primary BIA-ALCL tumor cells (*n* = 16), TLBR (*n* = 3), and cutaneous ALCL cell (*n* = 2) lines, PBMC purified from primary augmentation (*n* = 3), BIA-ALCL (*n* = 5), and contracture (*n* = 3) patients, and MT-4 cells following 72-h stimulation with LPS, PHA, SEA, and TSST-1, measured using an MTT assay. Each dot represents one cell line with six replicates and the mean SI is indicated by the horizontal line. Significance at * *p* < 0.05, ** *p* < 0.01, *** *p* < 0.001, **** *p* < 0.0001.

Patient-derived BIA-ALCL tumor cells and TLBR cell lines responded significantly more to LPS-induced stimulation compared to Staphylococcal superantigens SEA and TSST-1, *p* < 0.001, or to PHA, *p* < 0.01 (Figure 1).

In contrast, cutaneous ALCL cells responded significantly more to PHA and Staphylococcal superantigens SEA and TSST-1 than to stimulation with LPS, *p* < 0.01.

PBMC purified from healthy control patients, BIA-ALCL patients, and the transformed MT-4 cells responded significantly more to PHA compared with LPS, SEA, and TSST-1, *p* < 0.05 (Figure 1). In contrast, PBMC purified from capsular contracture patients responded significantly more to PHA and Staphylococcal superantigens than LPS, *p* < 0.05 (Figure 1). In both the cutaneous ALCL cell lines and PBMC from capsular contracture patients there was no significant difference between proliferative responses to PHA and Staphylococcal superantigens (*p* > 0.05).

The 95% confidence interval, *p* value, and partial omega squared (ω^2^*_p_*) values of each statistical comparison are listed in Table A4 and Table A5.

### 3.3. BIA-ALCL Cell Viability and Apoptosis in Response to Mitogen Stimulation

**Figure 2:**
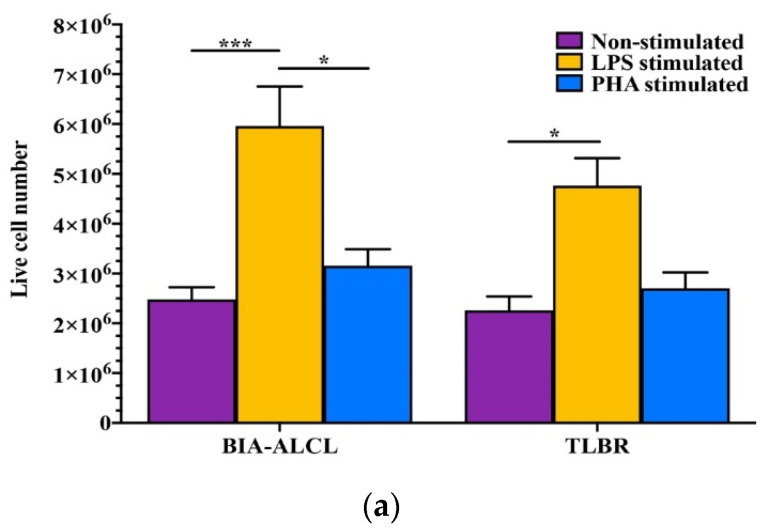

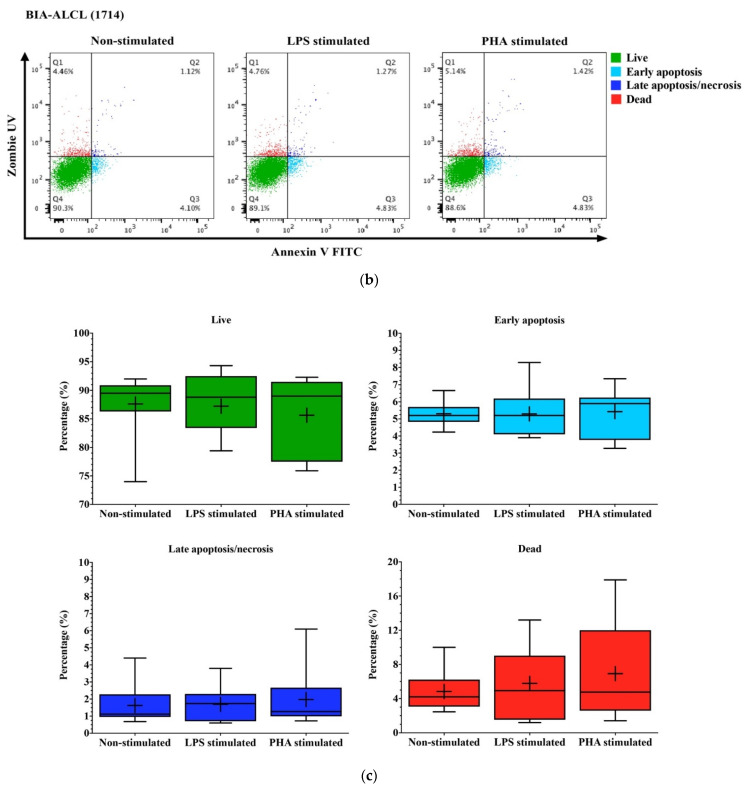
Viability and tolerance of BIA-ALCL cells to LPS- and PHA-induced proliferation as determined by flow cytometry. (**a**) Number of live BIA-ALCL (*n* = 11) and TLBR cells (*n* = 3). (**b**) Dot plots of BIA-ALCL tumor cells from patient 1714 stained with Zombie UV viability dye and FITC Annexin V showing live, early apoptotic, late apoptotic/necrotic, and dead cells. (**c**) The mean percentage of live, early apoptotic, late apoptotic/necrotic, and dead cells of BIA-ALCL (*n* = 11) and TLBR cells (*n* = 3). Error bars represent standard error of the mean. Box plot ‘+’ denotes the mean. Significance at * *p* < 0.05, *** *p* < 0.001.

LPS stimulation significantly increased BIA-ALCL (*n* = 11) and TLBR (*n* = 3) live cell number (*p* < 0.05, Figure 2a) but had no effect on cell viability or development of apoptosis. There was no difference found in the mean percentage of cells that were alive (BIA-ALCL 86–88%, TLBR 86–94%), dead (BIA-ALCL 5–7%, TLBR 2–8%), undergoing early apoptosis (BIA-ALCL 5%, TLBR 3–5%), or late apoptosis/necrosis (BIA-ALCL 1.6–2%, TLBR 0.5–1.3%) between stimulated and non-stimulated cells, as well as between BIA-ALCL cells and TLBR cell lines (*p* > 0.05) (Figure 2b,c).

### 3.4. Effect of Implant Surface Texture on BIA-ALCL Cell Proliferation

**Figure 3:**
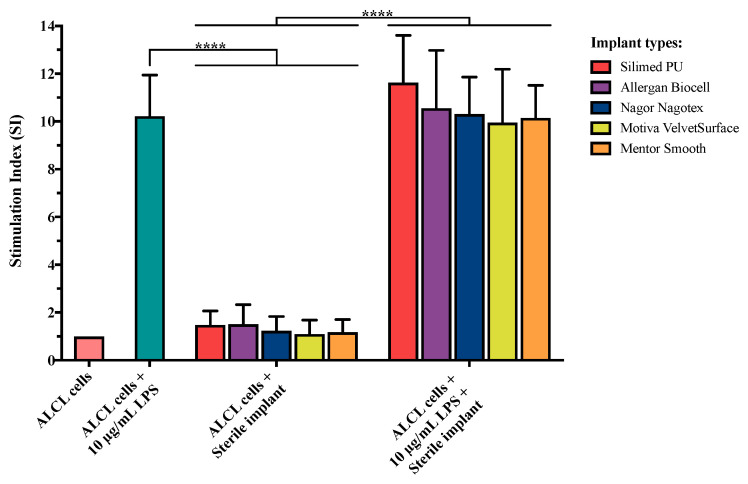
Proliferation response as stimulation index (SI) of BIA-ALCL tumor cells to various implant shell grades in the presence or absence of Gram-negative bacterial antigen LPS stimulation. Implants subjected to testing include High (Silimed PU), Intermediate (Allergan Biocell), Low (Nagor Nagotex), or Minimal (Motiva Velvet Surface and Mentor Smooth) grade. Error bars represent standard error of the mean of six replicates. Significance at **** *p* < 0.0001.

The presence of implant shells had no effect on BIA-ALCL cell proliferation. In the presence of implant shells alone graded as high/intermediate/low/minimal (Table A3), BIA-ALCL tumor cells failed to proliferate, with SI values of less than 1.5 (Figure 3). In contrast, LPS stimulation resulted in significant proliferation (*p* < 0.0001). Additionally, there was no potentiation of proliferation when different implant shell types were combined with LPS stimulation, *p* > 0.05 (Figure 3). This suggests the implant shells alone do not play a direct role in stimulation of BIA-ALCL tumor cells.

### 3.5. Higher TLR4 Expression in TLBR Cell Lines

**Figure 4:**
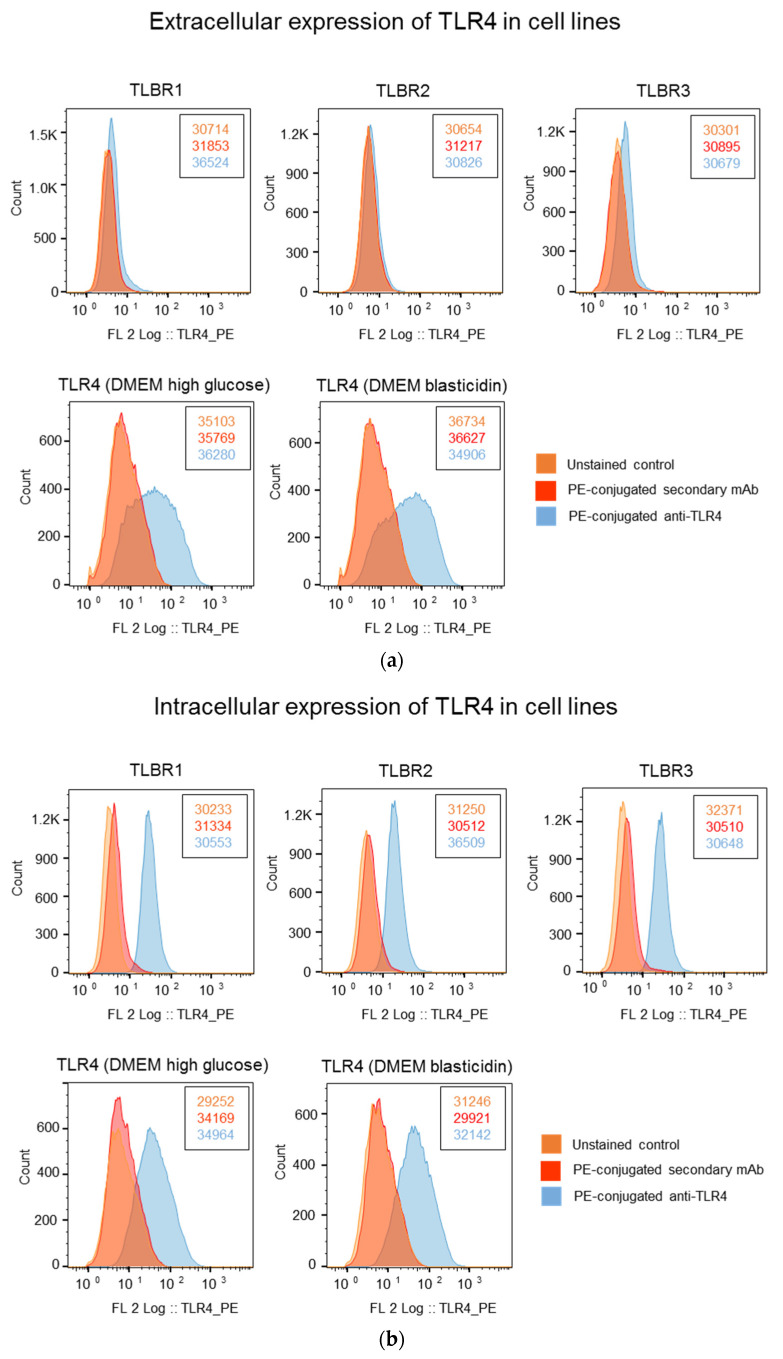
Analysis of extracellular expression (**a**) and intracellular expression (**b**) of TLR4 in BIA-ALCL cell lines and a positive control human TLR4 stable cell line, NBP2-26268, grown in DMEM ± Blasticidin in the presence or absence of LPS. The number of events (cell count) is shown next to the histograms.

TLR4 was detected in all three TLBR cell lines. Intracellular TLR4 was detected in 82% to 99% of TLBR cells regardless of the presence or absence of LPS in DMEM, but <5% of cells were surface stained (Figure 4). In the positive control human TLR4 stable cell line, approximately 18% to 24% of cells expressed surface TLR4 and 30% to 38% expressed intracellular TLR4 (Figure 4).

### 3.6. Effect of TLR4 Inhibition on LPS Stimulation of BIA-ALCL Cells

**Figure 5:**
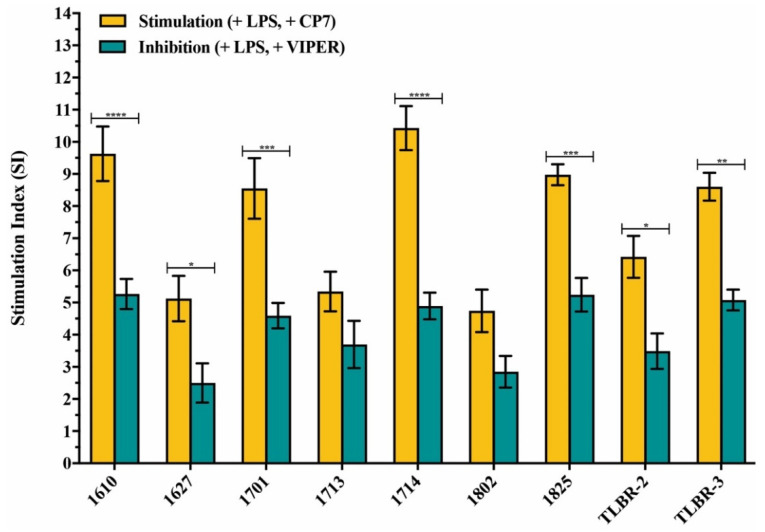
Inhibitory effect of the TLR4 inhibitor peptide VIPER on LPS-mediated TLR4 activation in BIA-ALCL cells measured by MTT. Values are the means ± standard error of the mean of triplicates. Significantly different at * *p* < 0.05, ** *p* < 0.01, *** *p* < 0.001, **** *p* < 0.0001.

The addition of TLR4 inhibitor peptide VIPER resulted in lower proliferative responses than the CP7 control peptide in all BIA-ALCL cells stimulated with LPS and this was significant in most BIA-ALCL cells (*p* < 0.05), except for patient numbers 1713 (*p* = 0.4253) and 1802 (*p* = 0.2546) (Figure 5).

**Figure 6:**
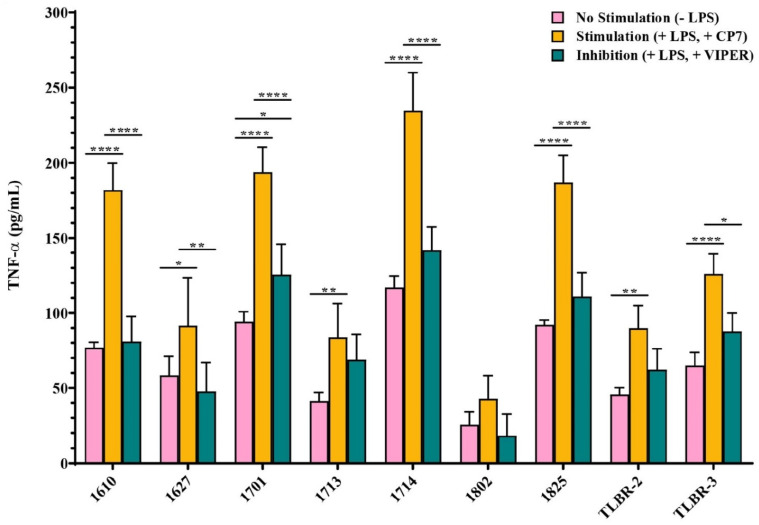
Inhibitory effect of the TLR4 inhibitor peptide (VIPER) on LPS-mediated TLR4 activation in BIA-ALCL cells as measured by ELISA measure of TNF-α. Negative (−LPS) and positive (+LPS) controls were also included. Values are the means ± standard error of the mean of triplicates. Significance at * *p* < 0.05, ** *p* < 0.01, **** *p* < 0.0001.

LPS stimulation increased production of TNF-α by BIA-ALCL cells, which was inhibited by addition of a TLR4 inhibitor peptide VIPER. The two TLBR cell lines and 6/7 BIA-ALCL patient cells produced significant amounts of TNF-α when stimulated with LPS, *p* < 0.05 (Figure 6). Addition of the TLR4 inhibitor peptide but not the CP7 control peptide inhibited LPS-induced TNF-α production in all BIA-ALCL tumor cells. There was significant inhibition in patients 1610, 1627, 1701, 1714, 1825, and in TLBR-3 (*p* < 0.05) (Figure 6).

## 4. Discussion

Our detailed analysis of the relationship between patient-derived BIA-ALCL primary tumor cells and tumor cell lines to a variety of bacterially derived antigens showed that there is a unique, proliferative response to the presence of Gram-negative bacterial LPS. This is in contrast to tumor cells from the phenotypically similar cutaneous form of ALCL, a T-cell leukemia cell line (MT-4), and PBMC derived from patients who have been diagnosed with capsular contracture and from those who have not been previously exposed to breast implants. Moreover, this response was absent in the PBMC from BIA-ALCL patients and, therefore, the response to LPS is a local tumor response and not a general systemic response.

In cutaneous ALCL cell lines and PBMC from capsular contracture patients, similar proliferative responses were found in the presence of Staphylococcal superantigens (SEA and TSST-1) and PHA. Indeed, the cutaneous ALCL cell lines used in this study were developed from a patient with a history of recurrent Staphylococcal skin infections while *Staphylococcus* spp. are frequently isolated from implants removed from patients with capsular contracture, suggesting that sensitization to Gram-positive antigens may have occurred in these patients.

The MTT assay is a colorimetric assay for measuring cell metabolic activity as an indicator of cell proliferation, viability, and cytotoxicity. Cell viability and apoptosis assays showed LPS stimulation significantly increased BIA-ALCL and TLBR live cell number but had no effect on cell viability or development of apoptosis. This suggests that the unique response of BIA-ALCL cells and TLBR cells’ lines to LPS stimulation measured by MTT assay is related to tumor cell proliferation.

The presence of breast implant shells of all surface grades 1–4 alone did not produce a proliferative response, nor did they show any potentiation of the LPS stimulation response, indicating that the implant shells alone do not play a direct role in stimulation of BIA-ALCL tumor cells. This suggests it is likely that the breast implant shell acts as a passive carrier for the growth of bacteria rather than acting as a pro-inflammatory stimulant.

These findings are consistent with the growing body of evidence around the epidemiology of BIA-ALCL that bacterial presence acts as a significant pro-inflammatory transformative driver in this lymphoma. The detection of a Gram-negative shift in the microbiome of BIA-ALCL tumor samples [7] is consistent with the results of our experiments. The global epidemiological distribution of cases reporting clusters of disease around a single surgeon experience [3] and the higher risk associated in implants with a higher surface area/roughness [3,5] reinforce the importance of bacterial contamination as a significant pathogenic mechanism [26].

The significance of bacterially driven malignant transformation in lymphoma and other cancers has become a topic of increasing worldwide attention with the availability of metagenomic analysis and better understanding of bacterial/host immune interactions [27]. In the case of BIA-ALCL, however, the infectious load is low-grade, indolent, and possibly polymicrobial with release of both Gram-positive and Gram-negative bacterial antigens into the peri-implant milieu. As infected breast implants cannot be treated successfully by antibiotic therapy, surgical removal can substitute for anti-microbial therapy. The complete regression of BIA-ALCL in patients with early-stage disease by surgical implant removal supports the hypothesis that removal of bacterial antigenic drivers can effectively treat the tumor [9].

In this study, we identified strong proliferative responses to LPS stimulation in patient-derived BIA-ALCL tumor cells and established BIA-ALCL cell lines. We found the response of BIA-ALCL cells to LPS is significantly dampened with the addition of a TLR4 inhibitor peptide, suggesting it is likely to be mediated via the TLR4 pathway. We also showed 82% to 99% of TLBR cells had cytoplasmic TLR4 staining. These findings are consistent with the previous study, which showed that IC14, an antibody that blocks CD14-mediated LPS binding to TLR4, can downregulate LPS-induced TNF-α [28], and support the previously described mechanism that interaction of LPS with TLR4 promote tumor growth [18]. Recent studies have shown that TLR4 has a unique property that it can travel between the surface plasma membrane and intracellular vesicles, such as endosomes and lysosomes [29], and these intra-cytoplasmic TLR4/LPS interactions are important determinants of ligand recognition and cellular signaling and can block induction of LPS-induced tolerance [30]. We detected intracellular TLR4 in most TLBR cell lines consistent with this pathway and that interaction of LPS via TLR4 directly activates lymphocytes to proliferate and survive as tumor cells in BIA-ALCL.

**Figure 7:**
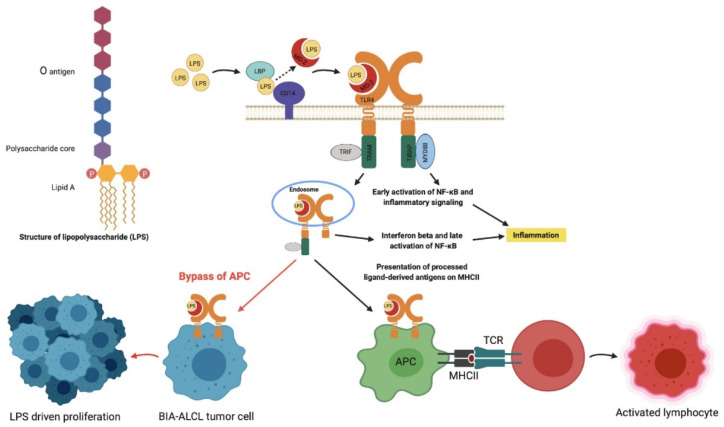
Proposed mechanism of lipopolysaccharide (LPS) activation in BIA-ALCL via TLR4 pathway. LPS activation of BIA-ALCL cells occurs through an alternative TLR4 pathway rather than T-cell receptor activation whereby the cells no longer require antigen presentation and processing by an APC. This complex is then able to directly activate T-cells, producing a downregulation of the immune response as a means to increase bacterial survival. LBP: LPS binding protein; MD2: myeloid differentiating protein 2; CD14: cluster of differentiation 14; TLR4: Toll-like receptor 4; TRIF: TIR domain-containing adaptor inducing interferon beta; TRAM: TRIF-related adaptor molecule; TIRAP: Toll-interleukin 1 receptor domain-containing adaptor protein; MyD88: myeloid differentiation primary response protein 88; NF-κB: nuclear factor kappa-light-chain-enhancer of activated B cells; MHCII: major histocompatibility complex 2; APC: antigen presentation cell; TCR: T-cell receptor. Figure created with BioRender.com, accessed on 26 November 2020.

TLRs are pattern recognition receptors in mammals that recognize damage-associated molecular patterns and pathogen-associated molecular patterns, including LPS [31]. The mechanism by which LPS triggers TLR4 is a complex process. LPS transfer is facilitated by LPS binding protein (LBP) and cluster of differentiation 14 (CD14) in the serum, which help LPS binding to the TLR4-myeloid differentiating protein-2 (MD2) complex [32]. TLR4 signaling can follow two different intracellular pathways: (1) MyD88-dependent pathway via TIRAP induces the transcription factor NF-kB resulting in the release of inflammatory cytokines and (2) MyD88-independent pathway via TRAM and TRIF leads to the release of type 1 interferons. This complex is able to bypass APC and directly activate T-cells, producing a powerful downregulation of the immune response and increased survival of bacteria [19] (Figure 7).

The response of BIA-ALCL tumor cells directly to LPS in the proliferation assays reveal the anaplastic cells no longer require processing and presentation of the antigen by an antigen presenting cell (APC) (Figure 7). Indeed, we demonstrated that although T-cell receptor rearrangements are universally apparent on deep-sequencing in BIA-ALCL, the T-cell receptor is non-functional [33]. This independence could result from de-differentiation of the tumor cell or derivation from an immature thymocyte precursor [34]. Whether the initial activation and transformation of a lymphoma precursor is triggered by LPS remains unanswered. The study of cellular interactions in early “benign” inflammatory peri-implant seromas may provide clues [35].

The role of LPS in both stimulating inflammation via the innate immunity pathway and dampening the host response via the adaptive immunity pathway is, therefore, unique and can both optimize bacterial survival and prolong host immune response and tissue damage [36]. It is probable that the presence of multiple bacterial species within bacterial biofilm in BIA-ALCL specimens [7] may also release different bacterial antigens from Gram-positive bacteria, which can further potentiate T-cell differentiation, proliferation, and malignant transformation. The presence of bacteria, coupled with a unique genetic (i.e., HLA variations for antigen processing) background of the host [37], would explain the relatively uncommon incidence of BIA-ALCL as it requires both bacterial presence and genetic susceptibility to cause ongoing immune activation and malignant transformation in susceptible hosts over time. A recent study of 11 consecutive patients with BIA-ALCL demonstrated a high frequency of JAK-STAT mutations and additional somatic mutations, as well as novel germline oncogene mutations [33].

The mechanism for peptide antigens as potentiators for driving the development of lymphoma has recently been elucidated in celiac disease [38]. In patients with refractory inflammation and unresponsive to a gluten-free diet (refractory celiac disease type II (RCDII)), there is a high rate of progression to small intestinal lymphoma. These patients have been shown to have an expanded lineage of innate intraepithelial lymphocyte (IEL) in the duodenum, which are the cells of origin for the lymphoma in these patients. These cells are thought to arise from an “early” T-cell or natural killer cell precursor and are sensitive to cytokines, such as IL-15, which cause selective expansion of premalignant IEL clones. In recent work, it has been shown that CD4+ T-cells activated by gluten result in cytokine-induced proliferation and survival of IEL. This proliferation induces mutations within the JAK-STAT pathway to establish presumably antigen-independent proliferation in time on the path toward true malignancy [37]. As an alternative or adjunctive pathway to malignancy, bacterial antigens could also drive T-cell malignancy in susceptible progenitor cells by induction of neighboring T-cells [38] as in cutaneous T-cell lymphoma. More recently, bacterially driven cross talk mediated by SEA causes upregulation of interleukin-17 with activation of STAT3 in neighboring T-cells to stimulate progression of malignant cutaneous T-cell lymphoma in a parallel of the mechanism in celiac-induced lymphoma [39].

The expanded role of the microbiome in carcinogenesis has also been reported in a number of epithelial tumors [32,40] including colorectal cancer [41,42], gastric cancer [43], breast cancer [44,45,46], prostate cancer [47], and oral cancer [48]. These studies show metagenomic differences and have also been taken into gnotobiotic (germ-free) mouse models to elucidate mechanistic pathways for transformation [49].

Understanding the mechanism(s) whereby a shift in bacterial populations harbored in the skin, gut, breast, prostate, oral cavity, and medical prosthetics influences the genesis of malignancy may provide us with a greater opportunity for prevention and future treatment of cancer.

## 5. Conclusions

The differential response of BIA-ALCL tumor cells to Gram-negative bacterial LPS support the hypothesis that bacterial antigens play a role in the pathogenesis of BIA-ALCL. This response is most likely mediated via TLR4 and represents an alternative pathway for bacteria to drive the pathogenesis of ALCL. Further work is ongoing to examine the potential for TLR blocking agents to minimize the risk of the development of this lymphoma. These data further support the surgical practice of minimizing the bacterial load on breast implants, an important goal for clinicians utilizing these implants in both aesthetic and reconstructive surgery.

## Data Availability

Dataset and metadata generated and/or analyzed during the current study are available from the corresponding author on request.

## Appendix A

**Table A1:** Clinical summary of the BIA-ALCL patients included in the study.

Scheme 1	Age at Diagnosis (Years)	Duration of Implant (Years)	Implant Type	Clinical Presentation	Stage ^1^	Treatment	Samples Analyzed
1610	38	13	Silimed PU ^2^	Seroma	1C	Surgery	Tumor cells
1612	45	5	Silimed PU	Seroma	1A	Surgery	Tumor cells
1618	51	14	Allergan Biocell	Seroma	1A	Surgery	Tumor cells
1626	45	10 1 2	Allergan Biocell Silimed PU Nagor	Capsular contracture followed by seroma	1A	Surgery	Tumor cells
1627	41	4 0.5	Allergan Biocell Allergan Biocell	Infection followed by revision and incidental mass	2A	Surgery	Tumor cells
1701	33	5	Silimed PU	Seroma	1A	Surgery	Tumor cells
1713	58	10	Allergan Biocell	Seroma	1A	Surgery	Tumor cells
1714	40	0.1 4 6	PIP ^3^ PIP Silimed PU	Contracture then seroma	1A	Surgery	Tumor cells
1715	31	5	Silimed PU	Seroma	1A	Surgery	Tumor cells
1802	29	9	Nagor	Seroma	1A	Surgery	Tumor cells
1803	57	8	Silimed PU	Seroma	1A	Surgery	Tumor cells, PBMC
1808	35	4	Nagor	Seroma	1A	Surgery	Tumor cells, PBMC
1810	37	3	Silimed PU	Seroma	1A	Surgery	Tumor cells, PBMC
1819	53	14.25	Mentor Siltex	Seroma	1A	Surgery	Tumor cells, PBMC
1821	52	9	Allergan Biocell	Seroma	1A	Surgery	Tumor cells, PBMC
1825	55	20	McGhan	Seroma	1A	Surgery	Tumor cells

^1^ Stage: BIA-ALCL Cancer staging as per 2019 NCCN guidelines; ^2^ PU: polyurethane; ^3^ PIP: poly implant prothèse.

**Table A2:** Clinical summary of established T-cell lymphoma breast cell lines.

Cell Line	Age at Diagnosis	Implant Type	Clinical Presentation	Treatment
TLBR-1	42	Nagor	Seroma	Surgery/Radiation therapy
TLBR-2	43	Allergan Biocell	Seroma	Surgery/Chemotherapy and radiation therapy—patient had recurrence and is deceased
TLBR-3	45	Allergan Biocell	Seroma	Surgery/Radiation therapy

**Table A3:** Classification and manufacturer of the various breast implant shell grades (adapted from Jones et al. [50]) used to determine their effects on BIA-ALCL tumor cell proliferation in the presence or absence of LPS stimulation.

Implant Type	Structure	Process	Surface Area	Roughness	Surface Type
Silimed Polyurethane (PU)		Polyurethane foam	High	High	4
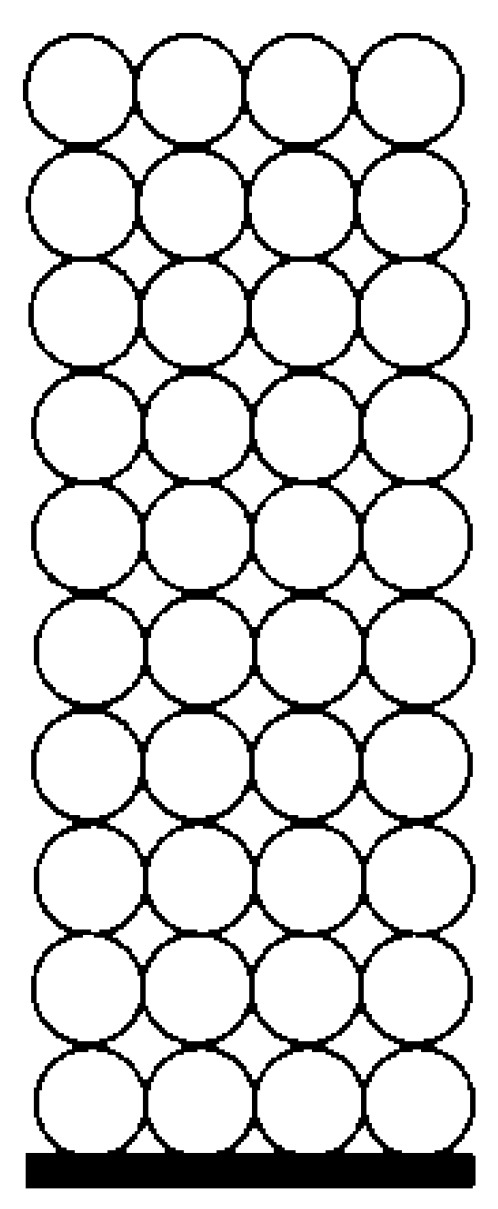
Allergan Biocell		Salt loss	Intermediate	Intermediate	3
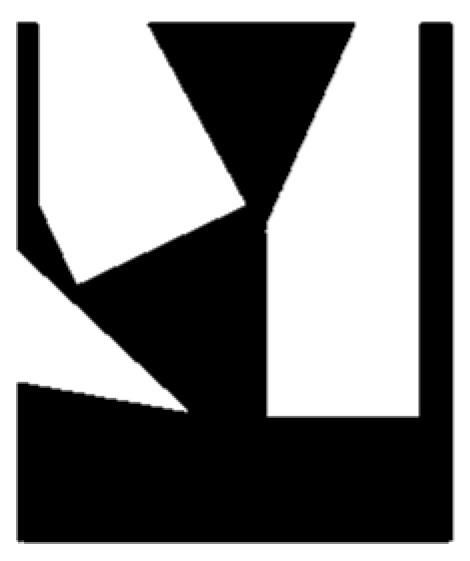
Nagor Nagotex		Salt loss	Low	Low	2
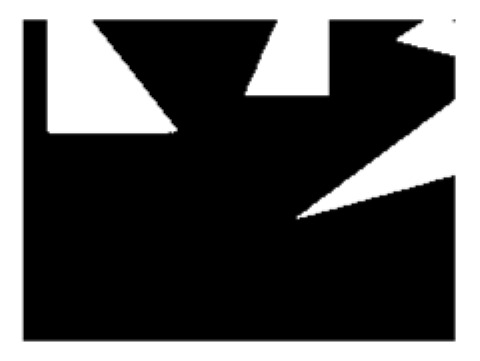
Motiva VelvetSurface		Nano	Minimal	Minimal	1
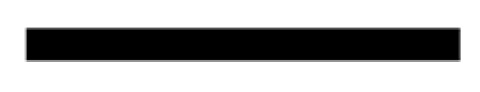
Mentor Smooth		Smooth	Minimal	Minimal	1

The implant surface grading diagram was reproduced with permission from Plastic and Reconstructive Surgery publisher, Wolters Kluwer Health, Inc. which has separate copyright and is not reproducible under the Creative Commons license.

**Table A4:** Tukey’s multiple comparisons’ test evaluating cell proliferation response to mitogenic/antigenic stimulation; 95% confidence interval for the mean difference and *p* values are shown.

Cells	Tukey’s Multiple Comparisons Test	95% Confidence Interval (CI) of Difference	Adjusted *p* Value
BIA-ALCL	LPS vs. SEA	1.490 to 4.093	<0.0001
	LPS vs. TSST-1	2.232 to 4.835	<0.0001
	LPS vs PHA	2.172 to 4.372	<0.0001
	PHA vs. SEA	−1.782 to 0.8207	0.7654
	PHA vs. TSST-1	−1.040 to 1.562	0.9519
	SEA vs. TSST-1	−0.7337 to 2.217	0.5510
TLBR	LPS vs. SEA	1.362 to 5.172	0.0001
	LPS vs. TSST-1	2.527 to 6.337	<0.001
	LPS vs PHA	0.9347 to 4.744	0.0011
	PHA vs. SEA	−1.477 to 2.332	0.9344
	PHA vs. TSST-1	−0.3125 to 3.497	0.1331
	SEA vs. TSST-1	−0.7400 to 3.070	0.3796
Cutaneous ALCL	PHA vs. LPS	1.329 to 5.995	0.0006
	SEA vs. LPS	0.6654 to 5.331	0.0064
	TSST-1 vs. LPS	2.303 to 6.969	<0.0001
	PHA vs. SEA	−1.669 to 2.997	0.8767
	PHA vs. TSST-1	−3.307 to 1.359	0.6908
	SEA vs. TSST-1	−3.971 to 0.6950	0.2598
MT-4	PHA vs. LPS	1.754 to 3.991	<0.0001
	PHA vs. SEA	2.115 to 4.351	<0.0001
	PHA vs. TSST-1	2.677 to 4.913	<0.0001
	LPS vs. SEA	−0.7575 to 1.479	0.8290
	LPS vs. TSST-1	−0.1960 to 2.040	0.1408
	SEA vs TSST-1	−0.5567 to 1.680	0.5497
BIA-ALCL PBMC	PHA vs. LPS	1.498 to 4.449	<0.0001
	PHA vs. SEA	1.337 to 4.288	<0.0001
	PHA vs. TSST-1	2.549 to 5.500	<0.0001
	LPS vs. SEA	−1.636 to 1.315	0.9917
	LPS vs. TSST-1	−0.4244 to 2.527	0.2479
	SEA vs TSST-1	−0.2635 to 2.688	0.1440
Capsular contracture PBMC	PHA vs. LPS	0.3294 to 4.139	0.0151
	SEA vs. LPS	0.7831 to 4.593	0.0023
	TSST-1 vs. LPS	1.118 to 4.928	0.0005
	PHA vs. SEA	−2.359 to 1.451	0.9230
	PHA vs. TSST-1	−2.694 to 1.116	0.6963
	SEA vs. TSST-1	−2.240 to 1.570	0.9667
Primary augmentation PBMC	PHA vs. LPS	1.507 to 5.317	<0.0001
	PHA vs. SEA	0.1812 to 3.991	0.0264
	PHA vs. TSST-1	0.6430 to 4.453	0.0042
	LPS vs. SEA	−3.231 to 0.5790	0.2669
	LPS vs. TSST-1	−2.769 to 1.041	0.6323
	SEA vs. TSST-1	−1.443 to 2.367	0.9192

**Table A5:** To obtain an unbiased measure of effect size, given the small number of cases for some of the cell lines tested, partial omega squared (ω^2^*_p_*) values were calculated.

Model	Effect Size (Partial Omega Squared, ω^2^*_p_*)	95% CI	Test Statistic	*p* Value
Mitogen type	0.0397	0.000 to 0.1323	*F*(3, 68) = 6.52	<0.001
Cell type	0.2217	0.0301 to 0.3558	*F*(5, 68) = 19.47	<0.001
Mitogen type × cell type	0.5177	0.2230 to 0.5810	*F*(15, 68) = 15.38	<0.001
